# Integrating Place Attachment into Management Frameworks: Exploring Place Attachment Across the Recreation Opportunity Spectrum

**DOI:** 10.1007/s00267-020-01292-7

**Published:** 2020-04-25

**Authors:** Christopher J. Wynveen, Ingrid E. Schneider, Arne Arnberger, Stuart Cottrell, Eick von Ruschkowski

**Affiliations:** 1 Baylor University, One Bear Place 97313, Waco, TX 76798 USA; 2 University of Minnesota, 301B Green Hall, 1530 Cleveland Avenue North, Saint Paul, MN 55108 USA; 3 Institute of Landscape Development, Recreation and Conservation Planning, Arne Arnberger University of Natural Resources and Life Sciences Vienna, Peter-Jordan-Straße 82 1190 Wien, Vienna, Austria; 4 Colorado State University, 1480 Campus Delivery, Fort Collins, CO 80523-1480 USA; 5 Alfred Toepfer Academy for Nature Conservation, Hof Möhr, 29640 Schneverdingen, Germany

**Keywords:** Experience use history, Management framework, Place identity, Place dependence

## Abstract

The importance of place in landscape management and outdoor recreation has been prominent in the literature since the 1970s. As such, calls to incorporate place into the management of parks, forests, and other protected areas exist. However, little work explores how place attachment may complement existing management frameworks. Hence, the purpose of this investigation was to explore levels of visitors’ place attachment intensity across the six classes of the Recreation Opportunity Spectrum (ROS). Survey data collected in North America and Europe indicated there was more similarity in place attachment intensities among areas classified toward the less developed end of the ROS, while greater variation existed among the more developed sites. Observing place attachment across all six ROS classes allowed for a deeper understanding of the correlation between place and the management framework.

## Introduction

The importance of place in outdoor recreation experiences has been prominent in the literature since the 1970s. Since Tuan (1977) posited that a setting becomes a place when it is endowed with meanings through lived experiences, authors have explored the interplay of place and recreation. Much of this research has involved “measuring the extent to which an individual values or identifies with a particular environmental setting” (Kyle et al. 2003, p. 250) to identify correlations among visitors’ attachment to outdoor recreation sites, attitudes, and recreation behaviors. For example, researchers have explored the connection between place attachment and the following: experience use history (EUH) and resource substitution (Eder and Arnberger 2012; Hammitt et al. 2004); activity type and involvement (Moore and Scott 2003); recreation motivations and support for management actions (Warzecha and Lime 2001); visitor conflict (Williams et al. 1992a); crowding and setting density (Kyle et al. 2004); sensitivity to resource conditions and pro-environmental behavior (Larson et al. 2018; Wynveen et al. 2013; Ramkissoon and Mavondo 2017); and attitudes toward fees (Kyle et al. 2003).

In light of the number and strength of connections between place attachment and outdoor recreation constructs, there have been several calls to incorporate place into the management of parks, forests, and other outdoor recreation sites (Morgan and Messenger 2009; Stewart et al. 2013). However, there has been little work to explore how place attachment may complement (or conflict with) existing management frameworks (e.g., the Recreation Opportunity Spectrum (ROS) or Visitor Experience and Resource Protection or Interagency Visitor Use Management Framework) (Beeco and Brown 2013). Current literature has neither undergone empirical testing nor been based on limited data from a relatively small set of setting types. For instance, Williams and Stewart (1998) posited that people can become attached to places from across the ROS, however minimal work explores if this is true or under what conditions it holds true. Furthermore, questions exist as to if the intensities of the subdimensions of place attachment (e.g., place identity and place dependence) that individuals report are similar across the ROS or do they diverge among categories of protected areas. Similarly, previous studies find an inverse relationship between area development and place attachment (e.g., Brown and Brabyn 2012), but it is unclear if this observation holds true across all ROS categories or only between those that represent the least and most developed.

Research on place attachment across the ROS needs to continue because, as Anderson and Fulton (2008) wrote, the “integration of place and place attachment into broader product-focused management frameworks is both a logical and necessary step for improving management of special places…” (p. 74). Further understanding people’s attachment to protected areas and how that attachment interacts with and can be incorporated into management frameworks, such as the ROS, may be useful in visitor management, encouraging pro-environmental behavior, and developing support for management actions. Hence, the purpose of this investigation was to explore levels of visitors’ place attachment intensity across the six classes of the ROS. The remainder of this paper documents this project that expands our understanding of the relationship between place attachment and the ROS and its implications for managers seeking to integrate place into existing management frameworks.

## Literature Review

### Place Attachment

Because “place attachment involves an interplay of affect and emotions, knowledge and beliefs, and behaviors and actions in reference to a place” (Altman and Low 1992, p. 5), measures of place attachment not only are an indication of the intensity of the human–place bond, but also abstractly provide insight on why individuals value a setting (Wynveen et al. 2010). Jorgensen and Stedman (2001) conceptualized place attachment as an attitude comprised of affective, cognitive, and conative components which develop in response to social interactions with a place and with others in a place (Stedman 2003). While building upon this conceptualization, others (Reineman and Ardoin 2018; Wynveen et al. 2012a) observed that both the physical characteristics of a setting and the lived experiences individuals have with others in the setting inform the reasons for which people become attached to a place and the intensity of that attachment.

To facilitate understanding of the intensity and, abstractly, the reasons for attachment, several researchers (e.g., Williams and Roggenbuck 1989; Kyle et al. 2004; Mesch and Manor 1998) have developed scales to measure place attachment. In the recreation and forest planning literatures, the most commonly used measure of place attachment has been Williams and Roggenbuck’s (1989) two-dimensional scale, designed to measure the degree to which an individual values or identifies with a setting through place identity and place dependence. The cognitive component, place identity, has been conceived of as a subcomponent of self-identity and, as such, refers to the person–place connection between a setting and personal identity (Proshansky 1978). Hence, people often use places to affirm their identity or express it to others (Twigger-Ross and Uzzell 1996). In doing so, the individual is communicating to themself and others “what should happen in [a place], what the setting is supposed to be like, and how the individual and others are supposed to behave in it” (Proshansky et al. 1983, p. 67).

The conative component of Williams and Roggenbuck’s scale is place dependence. Stokols and Shumaker (1981) first conceived of it as the association between a person and place based on the functional utility of the place. Later conceptualizations have specified that place dependence refers to “how well a setting serves goal achievement given an existing range of alternatives (‘how does this setting compare with others for what I like to do?’)” (Jorgensen and Stedman 2001, p. 234).

While many researchers have used Williams and Roggenbuck’s two-dimensional scale, others have suggested and/or used additional dimensions. For instance, Hammitt et al. (2004) measured dimensions labeled familiarity, belongingness, and rootedness. Acredolo (1982) conceptualized “familiarity” as an early bonding stage that involves perceptions of place knowing, security, and environmental preferences. “Belongingness” occurs when people feel an affiliation with or that they hold “membership” in a particular setting (Milligan 1998). As individuals become more attached, they may become “rooted” (Raadik Cottrell 2017). That is, Hay (1998) argued, they become possessive of the place and may stop seeking alternative settings to participate in the activities they enjoy. Others (e.g., Kyle et al. 2004) measured affective attachment and social bonding. “Affective attachment” is an emotional bond that people develop as they interact with a setting and interact with others in that setting (Jorgenson and Stedman 2001, Milligan 1998). Mesch and Manor (1998) suggested that “social bonding” is developed as social ties to a setting are created among individuals with shared experiences in a setting. Empirical support exists for the additional dimensions of place attachment (e.g., Hammitt et al. 2004; Ramkissooon et al. 2013; Wynveen et al. 2010; Wynveen et al. 2012a). But most studies have used exclusively or retained Williams and Roggenbuck’s measures of place identity and place dependence (Manning 2010). This practice is especially true in the international context. Williams and Roggenbuck’s bidimensional scale has been found reliable and valid across numerous contexts, whereas the more complicated multidimensional scales have not received as much use (especially outside of the North American and Australian contexts) or cross-cultural validation (Budruk 2010; Wynveen et al. 2017). Hence, the Williams and Roggenbuck’s scale was used for this investigation.

Beyond the conceptualization and measurement of peoples’ attachment to place, place research in natural environments has largely focused on relationships between place attachment and constructs important to the management and preservation of protected areas, such as national parks, forest reserves, and marine protected areas. For example, Ramkissoon and Mavondo (2017) identified a relationship between pro-environmental behavioral intentions and place attachment in Australian and Canadian national parks. Wynveen et al. (2013) reported similar results regarding marine protected areas in Australia and the United States. Other research has found a positive relationship between place attachment and quality-of-life, especially in the context of national parks (Ramkisson et al. 2018). In the tourism context, researchers have argued and found empirical support for a link between destination image (overall perception of a setting) and place attachment. For example, Jiang et al. (2017) observed that a perception of authenticity mediated the relationship between destination image and place attachment. Wynveen et al. (2010) argued that identifying and understanding the reasons why people become attached can be useful to marketing and educational communications needed to attract people to a protected area or encourage them to support conservation efforts and other management actions.

### Recreation Opportunity Spectrum

While a variety of recreation management frameworks exist (e.g., Visitor Experience and Resource Protection, Limits of Acceptable Change, and Visitor Impact Management), the ROS is the most widely known and applied (McCool et al. 2007) and “one of the most powerful inventory and analysis tools ever devised” (More et al. 2003, p. 9). The ROS allows managers and planners to consistently assess and then plan for a diversity of recreation opportunities based upon a combination of biophysical, social, and managerial attributes. The ROS is rooted in “experience-based setting management” (Floyd and Gramman 1997; Manfredo et al. 1983) where experiences result from recreation activities linked to specific settings: resource, social, and managerial. Theoretically, the ROS works from motivational theory as the experiences are sought out to satisfy motivations (Brown et al. 1978; Brown et al. 1979; Driver and Brown 1978).

The ROS has been refined for use in different U.S. regions (USDA Forest Service 1985; More et al. 2003) as well across the world such as in Norway, Japan, and Mexico (Kaltenborn and Emmelin 1993; Yamaki and Shoji 2004; Verden et al. 2008). Beyond geographic expansion, the idea of the ‘opportunity spectrum’ has also conceptually expanded to tourism (Butler and Waldbrook 2003) and water-based recreation (Haas et al. 2004).

The spectrum of opportunities emerges as planners inventory characteristics and then classify areas. The specific biophysical characteristics include: remoteness (distance from roads, trails), size (acres), and evidence of humans (impact of human modification); the social characteristics include the user density and encounters; and the managerial setting focuses on management noticeability and regimentation. The original spectrum focused on six land classes: primitive, semi-primitive nonmotorized, semi-primitive motorized, roaded natural, rural, and urban (USDA Forest Service 1985). With increased remoteness, fewer visitor encounters, and management interventions’ decreased noticeability, areas move toward the primitive end of the spectrum. An updated and refined spectrum focuses on: greater specificity on the urban end; replacing the ‘roaded natural’ with semi-developed natural and ‘rural’ with developed natural; and then refining ‘urban’ to highly developed with three subdivisions based on area size and facilities (More et al. 2003). As areas become more developed, they move into the semi-developed natural and developed natural classes. Semi-developed areas exhibit evidence of human modification of the environment that harmonizes with the natural environment and evidence of other visitors is prevalent, whereas developed areas are substantially modified and interaction among visitors is common. For example, More et al. (2003) suggest that areas larger than 5000 acres with few to no visitor encounters and little to no management presence are primitive, while small areas with frequent visitor contact in very developed areas are highly developed. The highly developed class is comprised of three subclasses (large natural, small natural, and facilities) that help delineate the class along considerations of geographic size, visitor experience, and physical characteristics. Both semi-primitive classes are characterized as medium to larger areas (greater than 2500 acres) predominantly appearing natural. The main difference between semi-primitive nonmotorized and semi-primitive motorized is the presence or lack of roads and motorized forms of recreation.

Research considering visitor characteristics within and across the ROS categories exists (e.g., Kyle et al. 2006; Wollmuth et al. 1985), as do select assessments of visitor behavior such as response to conflict (Schneider 2000), physical activity participation (Wilhelm Stanis et al. 2009), and response to terrestrial pest infestations (Schlueter and Schneider 2016). However, “the significance of specific places, which people come to know through experience, is not easily captured in resource and social assessments such as ROS” (Williams 2007, p. 31). Hence, researchers and managers need “to develop a more comprehensive understanding between setting and experience” (Williams 2007, p. 31). One place to begin improving our understanding is the intersection of place attachment and the ROS.

### Place Attachment and the Recreation Opportunity Spectrum

As the ROS, at least in conceptualization if not in practice, recognizes people–place relationships (Mitchell et al. 1993), consideration to place seems intuitive and useful. After a review of the literature, two observations can be made. First, numerous arguments can be made to explore place attachment and the ROS. For instance, Stedman et al. (2004) observed that reasons for people’s attachment to a place is related to interactions between ecological and socio-cultural factors. Given that the ROS inherently represents differing ecological and socio-cultural contexts, it is logical to propose that these varying contexts are associated with varying degrees of attachment. Alternatively, Kyle et al. (2004) investigated leisure activity involvement (i.e., the degree to which a person is devoted to an activity) which can reflect motivations for activity participation. They concluded that place attachment intensity was correlated with involvement and the natural setting type in which the activity took place. Again, as different socio-physical setting types which afford differing recreational activities are represented across the ROS, then it logically follows that visitors will develop varying degrees of attachment across the ROS classifications. More direct calls for the integration of place attachment and the ROS include Williams and Stewart (1998) who suggested people can become attached to places throughout the ROS classes. Furthermore, Anderson and Fulton (2008), among others (e.g., Kil et al. 2010; Kruger and Williams 2007; Stewart et al. 2013), called for the integration of place attachment into management frameworks. Second, only a handful of researchers have heeded these calls and used empirical methods to describe the association between place attachment and the ROS. Most researchers have done so only tangentially in that they have either used just one of the characteristic types included in the ROS (e.g., most often biophysical, but not social and managerial) as a proxy for the ROS class or examined the relationship among only a couple of the ROS classes. For example, Brown and Brabyn (2012) concluded that values for places differ across settings with different landscape characteristics (i.e., landcover, landform, presence of a water feature, and level of human development). More specific to the recreation context, Kil et al. (2012) observed that highly attached hikers in Florida’s wildland–urban interface preferred natural areas with little to no evidence of anthropocentric development. Others (e.g., Kainzinger et al. 2018; Warzecha and Lime 2001) observed greater differences in place dependence than place identity intensities ascribed to rivers with varying setting characteristics (i.e., number and degree of whitewater features). In contrast, Reineman and Ardoin (2018) found greater place identity than dependence among certain surf spots.

### Justification

Given the calls for the incorporation of place into management frameworks and the relative lack of empirical investigations incorporating place attachment and the ROS, this project explored levels of visitors’ place attachment intensity across the six classes of the modified ROS (More et al. 2003).

The working hypothesis was that place attachment intensity would vary across the ROS. Our logic for the hypothesis was based on the supposition that each class represents a range of physical (e.g., roads versus roadless) and social (e.g., more visitors to more developed sites and less visitors to less developed areas) attributes that afford differing lived experiences (e.g., types of recreational activities, group size, and type and frequency of intergroup interaction). While there is some overlap between attributes and lived experiences that take place among the classes, the ROS classification system was designed to afford a diversity of recreation opportunities (many of which can only be done in certain classes (e.g., wilderness backpacking is limited to the least developed class)) based upon a combination of biophysical, social, and managerial attributes (More et al. 2003). Varying combinations of physical and social attributes not only influence which ROS class is assigned to a particular protected area, these characteristics also influence individuals’ place attachment (Jorgensen and Stedman 2001). Furthermore, Wynveen et al. 2012 and others (e.g., Reineman and Ardoin 2018) made observations that supported the notion that the intensity of place attachment dimensions differs based on the physical and social attributes of a place. The shared importance of the physical and social attributes of a protected area to ROS classification and place attachment led to our suggestion that variations in context inherent to each class provide differing physical characteristics and afford differing lived experiences that result in different intensities of place attachment. This investigation extends previous research by being one of the first to observe levels of attachment to places across all six ROS classes, which allowed for a deeper understanding of the correlation between place and the management framework.

## Methods

To assess the association among place attachment and classifications across the ROS, we used data from identical survey items collected at seven sites spanning the six ROS classes. Each site was part of a larger series of investigations the research team conducted concerning landscape preference and recreation motivations. Hence, the sites were chosen to represent a range of physical and social characteristics. The areas ranged from larger undeveloped state parks to smaller urban forested areas with considerable development. Relevant to the current investigation is that all the survey instruments contained identical scales (e.g., Williams and Roggenbuck’s two-dimension place attachment scale) for the constructs of interest and all included a common core of research team members. Analysis began by assessing the place attachment scale measurement model across the seven sites and then tested for a correlation between place attachment and the ROS.

### Study Sites

Analysis began by identifying data from study sites that represented each of the six ROS classes. Because it is improbable to find a single protected area that contains all classes, we chose data collected from sites that represented at least one of the ROS classes. As the ROS is used across North America and to a lesser extent in Europe, multinational study sites were chosen. We acknowledge that use of multiple sites across North America and Europe may introduce error into the process. We attempted to limit that error by using a scale that has been deemed valid and reliable around the globe (when appropriately translated, as described below) (Lewicka 2011), and assessing the psychometric properties of the scale via confirmatory factor analysis and a measure of internal consistency. We also controlled for visitors’ frequency and tenure of visitation. Although error may still exist in regard to differing contexts, we chose to proceed with the analysis because this is the only data set to date that has amassed sufficient data points across all six ROS classes to conduct such analysis. We encourage future research to verify the results described below.

Table 1 identifies each site and the research team’s assessment of which class criteria was met for each of the ROS characterizations (i.e., settings, experience, remoteness, size, human criteria, social setting, and managerial) as outlined by More et al. (2003). Using a holistic view of the information, the research team assigned an ROS classification to each site (Table 2). U.S.-based sites included protected areas across the ROS. Colorado State Forest State Park is classified primitive with Minnesota’s Lake Bemidji State Park as an example of a semi-developed natural area on the ROS, and the highly developed (small natural subclass) Minnesota Historic Site and State Trail since adjacent to an urban state and national park. In Europe, study sites included Germany’s Harz National Park, which can be classified as semi-primitive nonmotorized. In addition, two developed natural areas (Danube Island and Prater Recreation Area) in Vienna, Austria, were included. Lastly, the Lobau, part of the Danube Floodplains National Park (also within the Vienna metropolitan area), was used and classified as semi-primitive motorized due to the fact that its narrow boundaries put most of the park in close proximity to major roadways, even though no public motorized roads exist within its borders (though there are several service roads). Across nations, the urban locations (i.e., Minnesota Historic Site and State Trail, Danube Island, Lobau, and Prater) were adjacent to major rivers and hosted a variety of activities in densely populated communities. For example, in Minnesota, the 29-acre Minnesota Historical Society area is nestled within a city park that receives more than 1.5 million annual visitors, a state park that receives 900,000 annual visitors, and a National Park Service property that hosts a culturally valued site with an estimated 130,000 visitors (Robbins Fenger, personal communication). Visitors hike, walk dogs, and enjoy the scenery for relaxation and physical activity. In the lesser developed protected areas, the locations were more rural and nestled among other federally or state-owned properties where visitors came for camping (i.e., Colorado State Forest State Park) or long hikes (i.e., Harz National Park, which does not allow overnight stays inside the park area) to enjoy the scenery and their own social group. Thus, the sites reasonably reflected the classes as described and detailed in the ROS.

**Table 1:** Sampling location characteristics: site size, annual visitation, and Recreation Opportunity Spectrum (ROS) classification

Site	Size ha (acre)	Visits in 1000 s		ROS class characterization^a^					
			Setting	Experience	Remoteness^b^	Size^b^	Human criteria	Social setting	Managerial
Minnesota Historical Site and State Trail	12 (29)	400	Highly developed	Highly developed	–	–	Highly developed	Highly developed	Highly developed
Danube Island	390 (963)	>4000	Developed natural	Developed natural	–	–	Developed natural	Highly developed	Developed natural
Prater	600 (1482)	4200							
Lake Bemidji State Park	687 (1700)	135	Semi-developed natural	Semi-developed natural	Semi-developed natural	–	Semi-developed natural	Semi-developed natural	Developed natural
Lobau, National Park	2300 (5683)	650	Semi-primitive motorized	Semi-developed natural	Semi-primitive motorized	Primitive	Semi-primitive motorized	Semi-developed natural	Developed natural
Harz National Park	25,700 (63,506)	1700	Semi-primitive nonmotorized	Semi-primitive nonmotorized	Semi-primitive motorized with primitive areas	Primitive	Semi-primitive nonmotorized	Semi-primitive nonmotorized	Semi-primitive nonmotorized
Colorado State Forest State Park	28,732 (71,000)	426	Semi-primitive motorized with Primitive areas	Primitive	Semi-primitive motorized with primitive areas	Primitive	Primitive	Primitive	Semi-primitive nonmotorized with primitive areas

^a^For operational definitions of each characterization setting criteria, see ref. More et al. 2003

^b^The ROS classification system does not have criteria for remoteness and size for the more developed end of the spectrum

**Table 2:** Sample size and response rate by site location and overall Recreation Opportunity Spectrum classification

Sampling Site	Location (data collection year, summers)	Recreation Opportunity Spectrum classification (sample size (*n*), response rate (rr))					
		Primitive	Semi-primitive nonmotorized	Semi-primitive motorized	Semi-developed Natural	Developed natural	Highly developed
Minnesota Historical Site and State Trail	Minneapolis, MN, USA (2018)						*n* = 152 rr = 50%
Danube Island	Vienna, Austria (2015)					*n* = 100 rr = 88%	
Prater	Vienna, Austria (2017)					*n* = 100 rr = 34%	
Lake Bemidji State Park	Bemidji, MN, USA (2014)				*n* = 228 rr = 74%		
Lobau National Park	Vienna, Austria (2015)			*n* = 200 rr = 34%			
Harz National Park	Altenau, Germany (2014)		*n* = 208 rr = 49%				
Colorado State Forest State Park	Walden, CO, USA (2014)	*n* = 200 rr = 65%					

### Instrument Design

Site visitors completed a multipage questionnaire addressing a range of recreational visitor perceptions relevant to the larger project. Beyond a series of demographic items, the survey instrument contained scales measuring place attachment and EUH. In addition, a variable representing ROS class was assigned to each site as described above.

Relying on classic place scales developed by Williams and colleagues (e.g., Williams and Roggenbuck 1989; Williams et al. 1992b; Williams and Vaske 2003), an eight-item scale (four items measure place dependence and four measure place identity) was used. All items were measured on a five-point agreement scale (i.e., 1 = strongly disagree, 3 = neither agree nor disagree, and 5 = strongly agree).

EUH was measured by asking respondents to indicate the number of visits in the last 12 months as well as total number of lifetime visits. A composite variable was then created using a cluster analysis procedure to identify natural groupings among the respondents (a low EUH and a high EUH clusters were identified). The EUH variable was used as a control variable because past research (e.g., Budruk et al. 2008; Eder and Arnberger 2012; Hammitt et al. 2004) has indicated that as EUH increases, so does place attachment. Given that the more developed sites in this investigation were closer to urban centers, there was a likelihood that those areas received greater repeat visitation from our respondents, in turn the visitors may report a higher attachment. Hence, the EUH variable was included in the analysis to minimize this possible effect.

To reduce error associated with translation and survey item interpretation, surveys were developed by native-language researchers (Budruk 2010), all surveys underwent professional translation with back-translation to check semantic equivalence (Wynveen et al. 2018), and items were removed from the analysis if they did not fit the measurement model across all data (Wynveen et al. 2017).

### Data Collection

To obtain a diverse and large sample, onsite data collection took place during summers (during peak visitation season as the sites were in northern climates) 2014 through 2018. Visitor intercepts procedures were similar across all sites. A stratified-cluster procedure was used to identify respondents at frequently visited sites (e.g., main access points, visitor centers, and trailheads) at each area. Both time of day and day of week were varied systematically to capture a diverse visitor segment (e.g., age gender, education, and experience with sites and activities). One adult, 18 years or older, per visitor group was asked to participate.

Total sample size was 1188 with 150 or more across sites representing each ROS class, with response rates ranging from 34% (i.e., Prater and Lobau) to 88% at Danube Island (Table 2). Across several of the sites, nonresponse reasons included not enough time or a lack of interest in participating in the study. Furthermore, refusal rates per activity type were collected at Harz National Park. Most of nonrespondents were hikers/walkers (93.6%), followed by dog walkers (4.5%) and bicyclists, joggers and Nordic walkers (1.9%). The lower response rate may have resulted in a nonresponse bias, however the nonresponse rates are within acceptable ranges and note a reported weak relationship between response rates and nonresponse bias (Johnston et al. 2017).

### Data Analysis

Questionnaire data were entered, initial descriptive analysis and assumptions (e.g., normality and assumption of variance) for each the statistical test were conducted using SPSS 25. To assess place attachment scale measurement, a confirmatory factor analysis and series of fit indices ensued in LISREL 10. We began by calculating the statistical power of the CFA Model (ten-items, two factors, *p* = 0.05) and determined that the sample size of 1188 with at least 150 representing each ROS classification resulted in a statistical power greater than 0.95 (which is greater than the recommend minimum of 0.80) (Soper 2020). This included assessing item factor loading and cross-loading between place identity and place dependence. For example, Brown (2006) suggested that, in applied research, items with factor loadings less than 0.40 be considered for removal from the model. Moreover, all items that loaded onto a single factor with above a factor loading of 0.4 were retained because the items included in the place attachment scale used are well established and have received significant empirical assessment. Regarding the overall model, the following criteria were used as indicators of acceptable fit for the place attachment measurement model: RMSEA ≤ 0.08 (Steiger and Lind 1980), CFI ≥ 0.95 (Hu and Bentler 1995), and Bentler and Bonnett’s (1980) suggested values of ≥0.95 as acceptable for the NNFI. In addition, Cronbach’s alphas were computed for each of the place attachment subscales.

To facilitate the comparison of place attachment among the ROS classes, place dependence and place identity scores were calculated by aggregating the corresponding items and calculating means. It is important to note that the choice to use an aggregate mean over a weighted mean calculation was based on precedence in the place literature (e.g., Hernandez et al. 2014; Jorgensen and Stedman 2001; Williams and Roggenbuck 1989, Williams and Vaske 2003) and the belief that this method better represents the operational definitions of place identity and place dependence. After testing for the appropriate assumptions, a set of analysis of covariances (ANCOVA) compared place identity and place dependence among the ROS classes, while controlling for EUH, with the appropriate post-hoc tests (Bonferroni) to identify the nature of any significant differences. Partial eta-squared statistics were calculated as a measure of effect size.

## Results

### Sample Characteristics

Visitor demographic composition was similar across most of the ROS classes. For instance, regarding gender, visitors to the semi-primitive nonmotorized, semi-primitive motorized, semi-developed natural, and highly developed areas were evenly split between males and females. In contrast, visitors to the primitive site were more frequently male (*n* = 118, 59%) and those to the developed site were more frequently female (*n* = 128, 64%) (*χ*^*2*^ = 23.51; *p* < 0.001). Similarly, the average respondent for most of the sites was middle aged (*M* = 46 years; SD = 15), with the exception of developed site visitors who, on average, were younger (*M* = 30; SD = 9) (*F*_df=5, 1168_ = 60.55; *p* < .001). Given that differences in age and gender did not vary systematically across the ROS classes in this data and the place literature has not identified a strong correlation between place attachment and gender or age (especially when controlling for EUH), we did not include these demographic variables as covariates in the remainder of the analysis. There were no differences in education level of respondents across the ROS classes (*F*_df=3_, _775_ = 1.73, *p* ≤ .16). Overall, about two-thirds (*n* = 519; 66.9%) indicated their highest level of education was a college or graduate degree.

Not surprisingly, significant differences were observed in both of the variables used to calculate the EUH variable among the ROS classes (visits to site in past 12 months: *F*_df=5_, _1023_ = 64.41, *p* < 0.001; visits ever to site: *F*_df=5_, _905_ = 172.55, *p* < 0.001). Generally, visitation in the past 12 months and total visitation both increased as the sites become more developed along the ROS. The only exception was that the semi-primitive motorized site was similar to the two developed categories in terms of total visitation. This is most likely an artifact of the fact that all three sites were near an urban center. Furthermore, as indicated in previous research, place identity and place dependence were significantly correlated (Pearson *r*) with each of the EUH variables (visits to site in past 12 months: was moderately correlated with place dependence (*r* = 0.33, *p* < 0.001); and weakly correlated with place identity (*r* = 0.19, *p* < 0.001); and visits ever to site: was moderately correlated with place dependence (*r* = 0.32, *p* < 0.001); and weakly correlated with place identity (*r* = 0.23, *p* < 0.001). Observations regarding the EUH-related items supported the decision to conduct the remainder of the analysis while controlling for EUH.

### Statistical Test Assumptions

Beyond sample characteristics, the data were also examined to ensure it met the assumptions necessary for CFA and ANCOVA. Because place dependence and place identity differed in the pooled sample, independent dimension analysis was conducted. Histogram and Q–Q plots indicated that the data were approximately normally distributed. Scatter plots and interaction tests indicated a homogeneity of regression slopes. Box plots and Cook’s distance test indicated that the data did not contain any influential outliers. Scatter plots also indicated linearity between EUH and each dimension of place attachment. Lastly, Levene’s test of equality of variances was significant for both place identity (*f*_5, 803_ = 8.48, *p* < 0.001) and place dependence (*f*_5, 800_ = 7.35, *p* < 0.001). However, due to the large sample size and relatively similar subsample sizes across the sites (i.e., ROS classes), we also examined the spread versus level plots (Rheinheimer and Penfield 2001; Steyn 2013). There was no discernable pattern in either, suggesting that we could proceed with the remainder of the analysis.

### Place Attachment Measurement

After calculating the demographic statistics, measurement of the two-dimensional place attachment scale ensued. Initial analysis revealed that two items needed to be removed due to low factor loadings (i.e., <0.40) and/or high cross-loading between the two dimensions. Removing poorly performing items increases discriminant validity between the dimensions and accounts for variations in the data that cannot be accounted for in other ways (e.g., language and cultural contextual differences that cannot be addressed by appropriate translation) resulting in model that more accurately represents the data (Brown 2006, Wynveen et al. 2017). Specifically, the item worded “The things I do at this recreation area I would enjoy doing just as much at a similar site” was removed from the measurement of place dependence and “This recreation area is very special to me” was deleted from place identity. The resulting place attachment measurement model (Table 3) fit the data well as indicated by acceptable fit indices (*χ*^*2*^_df=5_ = 10.49; RMSEA = 0.03; NNFI = 0.99; CFI = 0.99; *GFI* = 0.99). As a measure of internal consistency, Cronbach’s alpha was acceptable for place identity (*α* = 0.78) and borderline for place dependence (*α* = 0.67), given a general rule of thumb of at least 0.70 (Cortina 1993). Considering the CFA results, the long-established history of the place attachment scale used, that the place dependence measure only contains three items, and its close alpha, it was appropriate to retain the place dependence subscale for the analysis (Cortina). Using the resulting acceptable measurement model, the pooled sample (i.e., across all ROS classes) revealed visitors indicated a slightly greater place identity intensity (*M* = 3.50, SD = 0.94) than place dependence intensity *(M* = 3.10, SD = 0.92) (*t* = 17.37, *p* < 0.001), indicating a stronger cognitive identification with the protected area they visited than functional attachment.

**Table 3:** Place attachment scale confirmatory factor analysis results (data pooled across all sites, *n* = 1188)

Factor/Item	Mean	SD	λ	SE	Cronbach’s alpha
Place dependence	3.10	0.92			0.67
PD_1_ Doing what I do in this recreation area is more important to me than doing it in any other place	3.28	1.08	0.84	0.04	
PD_2_ I would not substitute any other recreation area for the type of recreation I do here	2.75	1.20	0.51	0.04	
PD_3_ No other place can compare with this recreation area	3.27	1.18	0.72	0.04	
PD_4_^a^ The things I do at this recreation area I would enjoy doing just as much a similar site	–	–	–	–	
Place identity	3.50	0.94			0.78
PI_1_ This recreation area means a lot to me	3.91	1.05	0.80	0.03	
PI_2_ Visiting this recreation area says a lot about who I am	3.15	1.21	0.59	0.04	
PI_3_^a^ This recreation area is very special to me	–	–	–	–	
PI_4_ I identify strongly with this recreation area	3.45	1.12	.75	.03	

Means based on a 5-point agreement scale, where 1 = strongly disagree, 3 = neither agree nor disagree, and 5 = strongly agree

CFA fit indices *χ*^2^_df=5_ = 10.49; RMSEA = 0.03; NNFI = 0.99; CFI = 0.99; GFI = 0.99

^a^PD4, PI3 were removed due to low factor loading and/or high cross-loading

### Place Attachment Across the ROS

The final analysis assessed whether and how place attachment varied across the ROS. The ANCOVAs compared each place attachment dimension among the ROS classes while controlling for EUH. Significant differences emerged for both place dependence and identity among the ROS classes (place dependence *f*_6, 806_ = 47.04, *p* < 0.001; place identify *f*_6, 809_ = 25.62, *p* < 0.001) (Table 4).

**Table 4:** ANCOVA: place attachment by ROS classification (controlling for experience use history)

Place attachment dimension	Mean (SD)						Effect of ROS class		
	Primitive	Semi-primitive nonmotorized	Semi-primitive motorized	Semi-developed natural	Developed natural	Highly developed	*f*	*p*	Partial *ƞ*^2^
Place dependence	2.94 (0.69)^a,b,c^	2.89 (0.88)^a,b,c^	3.84 (0.68)^d^	2.94 (0.73)^a,b,c^	2.62 (0.83)^e^	3.43 (0.99)^d^	49.19	<0.001	0.24
Place identity	3.57 (0.71)^a,b,c,d^	3.69 (0.99)^a,b,c,d^	3.99 (0.67)^a,b,d^	3.37 (0.86)^a,b,c^	2.97 (0.98)^e^	3.77 (0.93)^a,b,d^	30.54	<0.001	0.16

Model statistics: place dependence *f*_6, 806_ = 47.04, *p* < 0.001; place identify *f*_6, 809_ = 25.62, *p* < 0.001

Means based on a 5-point agreement scale, where 1 = strongly disagree, 3 = neither agree nor disagree, and 5 = strongly agree

^a–e^Means with different superscripts significantly different at *p* ≤ 0.05 (Bonferroni post-hoc tests)

#### Differences in place dependence across the ROS

Place dependence was significantly lower for the developed natural class (*M* = 2.62, SD = 0.83) than all other classes. The primitive (*M* = 2.94, SD = 0.69), semi-primitive nonmotorized (*M* = 2.89, SD = 0.88), and semi-developed natural (*M* = 2.94, SD = 0.73) classes were essentially equal and near the neutral-point of the response scale. Visitors to the semi-primitive motorized (*M* = 3.84, SD = 0.68) and highly developed (*M* = 3.43, SD = 0.99) classes reported significantly higher place dependence to the protected areas they visited. The effect size for place dependence was *ƞ*_*p*_^*2*^ = 0.24, indicating a weak-moderate effect.

#### Differences in place identity across the ROS

As compared with place dependence, there was slightly less variation in place identity among the ROS classes. As with place dependence, visitors to the developed natural class reported the significantly lowest level of place identity intensity (*M* = 2.97, SD = 0.98). In general, there were no significant differences in place identity intensity reported by visitors to the remaining classes (means ranged from 3.57 to 3.99 and standard deviations ranged from 0.67 to 0.99). The only exception was that there was a significant difference in place identity between the semi-primitive motorized (*M* = 3.99, SD = 0.67) and the semi-developed natural (*M* = 3.37, SD = 0.86) classes. Place identity’s effect size was *ƞ*_*p*_^*2*^ = 0.16, indicating a small effect.

## Discussion

This analysis explored levels of visitors’ place attachment intensity across the six classes of the modified ROS. We observed that although variations in place identity and place dependence across the six ROS classes existed, some nonlinear trends emerged. There seems to be a less developed/more developed dichotomy in that there was more similarity in place attachment intensities among less developed areas, while greater place dependence and place identity variation existed among the more developed sites. To understand these results, they must be interpreted considering the context of each ROS class (because each class provides differing physical and social characteristics that afford differing lived experiences) and the broader place attachment literature.

First, both the place identity and place dependence intensities were lowest for the developed natural area. In fact, for the respondents as whole, the low means indicated a lack of attachment. This is not to suggest that Williams and Stewart (1998) were wrong in their assertion that people can be attached to places throughout the ROS, because there are numerous individuals in the present sample that were attached to the areas that comprised the developed natural class. However, for many respondents, this finding suggests that Danube Island and Prater are not comprised of physical and social attributes that facilitate experiences sufficiently unique to foster place dependence. Nor did the sites encourage the development of place identity by affording the visitors the opportunity to incorporate these protected areas into their identity. Perhaps as these areas are better integrated in the urban fabric, there are several alternative developed natural substitutes (i.e., place with similar setting characteristics) convenient for the respondents. Hence, the protected areas do not provide a resource that visitors depend on to enjoy the types of activities they participate in or there is not enough distinctiveness to these areas for them to be used by the individual to express or confirm their identity, an inference that is supported by past literature on place attachment, EUH, and substitution (Hammitt et al. 2004; Wynveen et al. 2008). As both areas are within the Vienna metropolitan area, it is likely that they serve as substitutes for one another. Alternatively, one might argue that developed natural areas are relatively common to most of North Americans and Europeans, who often live near densely populated areas, that they are too ubiquitous to engender a higher intensity of attachment. Specific, in-depth, further research should be undertaken to understand the phenomenon observed at these protected areas. Research should also investigate whether similar relative levels of place attachment occur at other developed protected areas, with similar physical and social attributes, within urban settings.

While the commonplace landscape of developed natural areas may explain the associated place attachment levels, it seems counterintuitive to our observation that the highly developed area had one of the highest levels of place dependence and place identity. This site is situated directly between a highly developed metropolitan park rated as the number one attraction in the metropolitan area by TripAdvisor and a highly visited state park (facts that speak to the importance of social attributes of the site), as well as being adjacent to the most frequently visited dog park in the Twin Cities of Minneapolis and St. Paul Minnesota. Thus, the trail affords access to a variety of adjacent experiences that may meld in visitor experience recall. Also, regarding the physical attributes of the setting, the MHS site is unique in that while highly developed as dictated by size and distance to roads, it affords escape from urban noise pollution if one follows the trails to the river. Notably, highly developed protected areas are often designed with a more singular purpose to highlight a significant cultural or natural resource (i.e., unique social and physical attributes). In this instance, the Minnesota Historic Site is historically important for indigenous peoples and Europeans and also provides unique access to the federally protected Mississippi River. Hence, the site’s importance spans generations and cultures. As such, the place attachment to the Minnesota Historic Site and State Trail is similar to past research which has observed that places that contain a unique natural resource and are culturally significant can lead to place attachment (Wynveen et al. 2012). Similarly, accessibility and historically important sites (Gunderson and Watson 2007) and places that facilitate recreational enjoyment (Bricker and Kerstetter 2002) have been associated with higher place attachment intensities.

Previous research has observed a negative correlation between increased level of development and place attachment (Brown and Brabyn 2012; Kil et al. 2012). The present data were less clear in this regard. For instance, there was not an increase in place identity intensity moving through the ROS classes from semi-primitive motorized to semi-primitive nonmotorized to primitive. This suggests that for these respondents the correlation between ROS class and place attachment is sufficient once a protected area exhibits physical and social characteristics that situate it on the primitive half of the spectrum (i.e., no gains were made by becoming even more primitive). One explanation, supported by similar observations by Kyle et al. (2004), may be that given the geographical size requirements of the three primitive classes (i.e., >2500 acres), there is an accompanying decrease in encounters with other visitors and management regulation via interaction with rangers and other staff. Social interaction at this level is so small that any subsequent decrease from class to class is imperceptible. Thus, it does not influence place identity. Furthermore, besides size, the other perceptible difference among these classes is the presence of physical attributes that represent the built environment, such as roads (and possibly vehicles). However, even in a semi-primitive motorized area, a visitor may usually easily avoid roads and vehicles, if they choose. Hence, the respondents may not have perceived the proximity of roads as a distinguishing characteristic at the semi-primitive sites and therefore it was not correlated with their place identity. Similarly, one might argue that the physical attributes (e.g., predominately natural setting and relative size of the protected areas) in these classes facilitated recreational activities that allowed respondents to express/confirm their identity as a hiker, nature lover, backpacker, outdoors person, et cetera, that is not afforded by more developed sites that have different physical and social attributes that encourage different lived experiences.

The last noteworthy result was that place dependence differed from place identity, in that for place dependence the semi-primitive motorized class was significantly greater than the primitive and semi-primitive nonmotorized classes (as well as the semi-developed natural and developed natural classes). While this corresponds with recent work by Kainzinger et al. (2018), who observed differences in place dependence (but not place identity) in four river settings, this is contrary to previous research that observed greater place dependence intensity associated with less developed areas (Warzecha and Lime 2001; Wynveen et al. 2008). Given the paucity of the literature on place attachment and the ROS, it is difficult to explain this apparent contradiction. One possibility is that the present finding is an artifact of the location of the protected areas that represented the two more primitive classes. Both Colorado State Forest State Park and Harz National Park are located in regions that include other state and national protected areas that have similar biophysical, social, and managerial attributes and afford similar types of recreational activities. Similar to the argument made by Reineman and Ardoin (2008) concerning California surfers with multiple destination options for their activity, our respondents may not value the functional utility of Colorado State Forest State Park and Harz National Park, as opposed to the peri-urban Lobau. This is because Lobau is a considerable distance from other protected areas that would be classified on the primitive end of the ROS. In addition, the Lobau is the only larger attractive recreation area to the east of Vienna, which is heavily visited by local residents. More research is needed to understand the present findings and those in the limited existing literature.

### Limitations and Future Research

In addition to limitations already outlined in the methods section, the data represent a wide range of contexts (e.g., urban and rural, North American and European) and two languages (English and German). Hence, results need to be interpreted in light of the fact that the data were collected at multiple sites, and some error stemming from contextual differences beyond those represented by the ROS may still exist, although as indicated throughout this paper, this error was controlled for where possible (e.g., survey instrument translation).

Furthermore, the samples were drawn from a general population of recreational visitors to each site. Therefore, inherent to this sampling method, the effect of visitor type (e.g., tourists, locals, and backcountry enthusiasts) or recreation experience preference (e.g., mode of experience preference, recreation specialization, and recreational) on the results was beyond the scope of this analysis. Also, regarding visitor characteristics, the visitor base is likely changing and future research needs to address this. The majority of the sample identified as white, non-Hispanic. As the population in the United States and Europe diversifies, understanding diverse perspectives on place attachment and recreation preference is important.

Lastly, while this project assessed place attachment across a range of protected area settings, it did not assess place attachment development or changes across time. As such, understanding the relative speed at which place attachment develops across ROS categories and its origin would be of interest (for a discussion, see di Massoa et al. 2019). This information may also be useful in understanding visitors’ affective, cognitive, and conative responses to natural and anthropocentric triggered landscape change. Albrecht (2005) suggested that severe landscape change can cause solastalgia (distress caused by environmental change). For example, Schneider et al. (2019) found more than 70% of visitors experienced potential displacement as bark beetle infestations changed landscapes: what might such changes do to place attachment and the association between attachment and the ROS? Further, in an increasingly mobile society, the notion of multiple place attachments and assumptions of sedentary attachments must be considered (Di Masso et al. 2019).

In sum, future research regarding place attachment and the ROS should include focused efforts to reach out to visitors representing a range of activity preferences, racial and ethnic categories, and other demographics of interest. Researchers should explore the association over time and outside of the North American and European contexts. They may also consider the effect of environmental change on place attachment across the ROS.

## Management Implications and Conclusion

The present findings confirm that there is an association between place attachment and the ROS. The interpretation of the data suggested that the association may, in part, be an artifact of the varying physical and social attributes of the settings that are representative of each ROS class. In turn, these attributes afford different lived experiences resulting in varying place identity and place dependence intensities among visitors. As such, this work supports the ideas and research of Williams and Stewart (1998), Brown and Brabyn (2012), Kil et al. (2010, 2012), and others. These findings extend the previous literature by describing this relationship across all six ROS classes. Specifically, we observed that although there is an association between the constructs, it is not linear. It is likely that the sources of the nonlinear relationship are variables that we did not measure, but that exert a moderating effect on place attachment across the ROS. As suggested throughout this discussion, future research should identify other constructs of interest that influence this association.

Like other authors, such as Anderson and Fulton (2008) and Stewart et al. (2013), the integration of place attachment into the ROS and other management frameworks is one of our suggestions. While these results suggested that it is not as easy as asserting visitors prefer this class or that class, results do indicate a greater need to understand the role the human–place bond plays in the settings and activities people choose to visit and participate in. Echoing the calls of others, this may require researchers and managers to develop a greater understanding of the place meanings ascribed to protected areas (Wynveen et al. 2012) and the recreation experience preferences visitors have (Budruk and Wilhelm Stanis 2013; Raadik et al. 2010), in order to use this information to further recognize the reasons for attachment and how the intensity of place attachment varies across the ROS.

Finally, the results further contribute to the discussion of how to incorporate place attachment into management frameworks. Williams and Stewart (1998) outlined four steps for managers to follow to do so. In the context of the ROS and in light of the present results, managers should know and use local place names when working with stakeholders to (re)classify protected areas into one of the ROS classes. Doing so allows incorporation of place attachment, especially the reasons for the attachment, to be included in ROS planning as local place names often reflect the intensity of and reason for attachment to a specific setting. Second, use of these local terms and reasons for attachment to communicate management plans and objectives (Wynveen et al. 2010). For example, local names and reasons for attachment can be used to prime messaging designed to encourage pro-environmental or other behaviors or engage visitors in environmental education that will help maintain an area’s social and physical characteristics within a specific ROS class. Both of the first two suggestions also communicate an understanding of the importance of the social attributes of a setting, in conjunction with the physical attributes, to stakeholders. This communication is particularly important given observations indicated varying degrees of attachment across the ROS and that previous research has indicated that communicating in local terms builds trust between stakeholders and managers and increases stakeholder support for management decisions (Kyle et al. 2003; Wynveen et al. 2010). For example, visitors to less developed settings with greater place attachment may be more likely to support conservation-minded actions and programs (Ramkissoon and Mavondo 2017; Wynveen et al. 2013). Third, the confirmation of differing levels of place attachment among the ROS classes highlights the need for managers to understand the differences in and the reasons for the attachment across the ROS classes they manage. In other words, understanding the importance of the social and physical attributes of a protected area that allow for lived experiences important to stakeholders. Doing so will help managers understand the “politics of place” that occur when stakeholder groups hold differing place attachment intensities. Similarly, as suggested by Jiang et al. (2017) knowledge of setting attributes that lend themselves to specific ROS classification and increased place attachment intensity may also be useful to tourism marketing strategies that seek to communicate information about destination image and authenticity of a protected area to potential visitors. Lastly, managers should pay close attention to places that have different (or opposing) reasons for attachment to different groups (Williams and Stewart 1998), especially when place attachment intensity is high among all stakeholders involved. This knowledge can be helpful to identify and mitigate stakeholder conflict.

In conclusion, this was one of only a few studies that has sought to empirically explore the association between ROS and place attachment. To our knowledge, it is the first to assess place attachment among all six ROS classes. The data indicated that place attachment and ROS are associated. Hence, the findings suggest a need to further seek to understand how these constructs interact with one another and to identify other variables that influence this relationship. Lastly, we believe these results further the call for the incorporation of place attachment into management frameworks.

## References

[CR1] Acredolo LP (1982). The familiarity factor in spatial research. N. Dir Child Dev.

[CR2] Albrecht G (2005). Solastalgia: a new concept in health and identity. Philos Act Nat.

[CR3] Altman I, Low S, Altman I, Low S (1992). Place attachment: a conceptual inquiry. Place attachment.

[CR4] Anderson DH, Fulton DC (2008). Experience preferences as mediators of the wildlife related recreation participation: place attachment relationship. Hum Dimens Wildl.

[CR5] Beeco JA, Brown G (2013). Integrating space, spatial tools, and spatial analysis into the human dimensions of parks and outdoor recreation. Appl Geogr.

[CR6] Bentler P, Bonett D (1980). Significance tests and goodness of fit in the analysis of covariance structures. Psychol Bull.

[CR7] Bricker KS, Kerstetter DL (2002). An interpretation of special place meanings whitewater recreationists attached to the South Fork of the American River. Tour Geogr.

[CR8] Brown TA (2006). Confirmatory factor analysis for applied researchers.

[CR9] Brown P, Driver B, Bruns D, McConnell C (1979) The outdoor recreation opportunity spectrum in wildland recreation planning: Development and application. First Annual National Conference on Recreation Planning and Development: Proceedings of the Specialty Conference 2. Society of Civil Engineers, Washington D.C. 1–12

[CR10] Brown P, Driver B, McConnell C (1978) The opportunity spectrum concept in outdoor recreation supply inventories: Background and application. Proceedings of the Integrated Renewable Resource Inventories Workshop. Gen. Tech. Rep. RM-55. USDA Forest Service, Rocky Mountain Research Station, Fort Collins, CO, pp 73–84

[CR11] Brown G, Brabyn L (2012). An analysis of the relationships between multiple values and physical landscapes at a regional scale using public participation GIS and landscape character classification. Landsc Urban Plan.

[CR12] Budruk M (2010). Cross-language measurement equivalence of the place attachment scale: a multigroup confirmatory factor analysis approach. J Leis Res.

[CR13] Budruk M, Wilhelm Stanis S (2013). Place attachment and recreation experience preference: a further exploration of the relationship. J Outdoor Rec Tour.

[CR14] Budruk M, Wilhelm Stanis S, Schneider IE, Heisey J (2008). Crowding and experience use history: a study of the moderating effect of place attachment among water-based recreationists. Environ Manag.

[CR15] Butler R, Waldbrook L (2003). A new planning tool: The tourism opportunity spectrum. J Tour Stud.

[CR16] Cortina JM (1993). What is coefficient alpha? An examination of theory and applications. J Appl Psychol.

[CR17] Cottrell JR (2017). Island community: identity formulation via acceptance through the environment in Saaremaa, Estonia. Isl Stud J.

[CR18] Di Masso A, Williams DR, Raymond CM (2019). Between fixities and flows: navigating place attachments in an increasingly mobile world. J Environ Psycho.

[CR19] Driver B, Brown P (1978). The opportunity spectrum concept in outdoor recreation supply inventories: a rationale. Gen Tech Rep RM-55.

[CR20] Eder R, Arnberger A (2012). The influence of place attachment and experience use history on perceived depreciative visitor behavior and crowding in an urban national park. Environ Manag.

[CR21] Floyd MF, Gramman JH (1997). Experience-based settings management: Implications for market segmentation of hunters. Leis Sci.

[CR22] Gunderson K, Watson A (2007). Understanding place meanings on the bitterroot national forest, Montana. Soc Nat Resour.

[CR23] Haas G, Aukerman R, Lovejoy V, Welch D (2004). Water recreation opportunity spectrum (WROS) user’s guidebook.

[CR24] Hammitt WE, Backlund EA, Bixler RD (2004). Experience use history, place bonding and resource substitution of trout anglers during recreation engagements. J Leis Res.

[CR25] Hay R (1998). A rooted sense of place in cross-cultural perspective. Can Geogr.

[CR26] Hernandez B, Hidalgo MC, Ruiz C, Manzo LC, Devine-Wright P (2014). Theoretical and methodological aspects of research on place attachment. Place attachment: Advances in theory, methods and applications.

[CR27] Hu L, Bentler PM, Hoyle RH (1995). Evaluating model fit. Structural equation modeling: concepts issues and applications.

[CR28] Jiang Y, Ramkissoon H, Mavondo FT, Feng S (2017). Authenticity: the link between destination image and place attachment. J Hosp Mkt Manag.

[CR29] Johnston RJ, Boyle KJ, Adamowicz W (2017). Contemporary guidance for stated preference studies.. J Assoc Environ Resour Econ.

[CR30] Jorgensen BS, Stedman RC (2001). Sense of place as an attitude: Lakeshore owners’ attitudes toward their properties. J Environ Psychol.

[CR31] Kainzinger S, Arnberger A, Burns RC (2018). An examination of whitewater boaters’ place attachment and specialization in four different river settings. Environ Manag.

[CR32] Kaltenborn BP, Emmelin L (1993). Tourism in the high North: management challenges and recreation opportunity spectrum planning in Svalbard, Norway. Environ Manag.

[CR33] Kil N, Holland SM, Stein TV (2010). Improving the management of natural resource recreation areas through understanding place-attached visitor segments. J Park Recreat Admi.

[CR34] Kil N, Stein TV, Holland SM, Anderson DH (2012). Understanding place meanings in planning and managing the wildland–urban interface: the case of Florida trail hikers. Landsc Urban Plan.

[CR35] Kruger LE, Williams DR (2007) Place and place-based planning. In Kruger LE, Mazza R, Lawrence K (eds) Proceedings: National workshop on recreation research and management. Gen Tech Rep PNW-GTR-698. USDA Forest Service, Pacific Northwest Research Station, Portland, OR, pp 83–88

[CR36] Kyle GT, Absher J, Graefe AR (2003). The moderating role of place attachment on the relationship between attitudes toward fees and spending preferences. Leis Sci.

[CR37] Kyle GT, Absher J, Hammitt WE, Cavin J (2006). An examination of the motivation-involvement relationship. Leis Sci.

[CR38] Kyle GT, Bricker K, Graefe AR, Wickham T (2004). An examination of recreationists’ relationships with activities and settings. Leis Sci.

[CR39] Kyle GT, Graefe AR, Manning R, Bacon J (2004). Effects of place attachment on users’ perceptions of social and environmental conditions in a natural setting. J Environ Psychol.

[CR40] Kyle GT, Mowen AJ, Tarrant M (2004). Linking place preferences with place meaning: an examination of the relationship between place motivation and place attachment. J Environ Psychol.

[CR41] Larson LR, Usher LE, Chapmon T (2018). Surfers as environmental stewards: Understanding place-protecting behavior at Cape Hatteras National Seashore. Leis Sci.

[CR42] Lewicka M (2011). Place attachment: How far have we come in the last 40 years?. J Environ Psychol.

[CR43] Manfredo MJ, Driver B, Brown P (1983). A test of concepts inherent in experience based setting management for outdoor recreation areas. J Leis Res.

[CR44] Manning RE (2010). Studies in outdoor recreation: Search and research for satisfaction.

[CR45] McCool SF, Clark RN, Stankey GH (2007). An assessment of frameworks useful for public land recreation planning. Gen Tech Rep PNW-GTR-705.

[CR46] Mesch GS, Manor O (1998). Social ties, environmental perception, and local attachment. Environ Behav.

[CR47] Milligan MJ (1998). Interactional past and potential: the social construction of place attachment. Symb Interact.

[CR48] Mitchell MY, Force JE, Carroll MS, McLaughlin WJ (1993). Forest places of the heart. incorporating special spaces into public land management. J For.

[CR49] Moore RL, Scott D (2003). Place attachment and context: comparing a park and a trail within. Sci.

[CR50] More TA, Bulmer S, Henzel L, Mates AE (2003). Extending the recreation opportunity spectrum to nonfederal lands in the Northeast: Gen Tech Rep NE-309. An implementation guide.

[CR51] Morgan M, Messenger B (2009). Using an activity and place-based typology to explain visitor motivations. J.

[CR52] Proshansky HM (1978). The city and self-identity. Environ Behav.

[CR53] Proshansky HM, Fabian AK, Kaminoff R (1983). Place-identity: Physical world socialization of the self. J Env Psych.

[CR54] Raadik Cottrell J (2017). Island community: identity formulation via acceptance through the environment in Saaremaa, Estonia. Island Stud J.

[CR55] Raadik J, Cottrell SP, Fredman P, Ritter P, Newman P (2010). Recreational experience preferences: application at Fulufjället National Park, Sweden. Scand J Hosp Tour.

[CR56] Ramkissoon H, Mavondo FT (2017). Proenvironmental behavior: critical link between satisfaction and place attachment in Australia and Canada. Tour Anal.

[CR57] Ramkissoon H, Mavondo FT, Uysal M (2018). Social involvement and park citizenship as moderators for quality-of-life in a national park. J Sustain Tour.

[CR58] Ramkissoon H, Smith LDG, Weiler B (2013). Testing the dimensionality of place attachment and its relationships with place satisfaction and pro-environmental behaviours: a structural equation modelling approach. Tour Manag.

[CR59] Reineman DR, Ardoin NM (2018). Sustainable tourism and the management of nearshore coastal places: place attachment and disruption to surf-spots. J Sustain Tour.

[CR60] Rheinheimer DC, Penfield DA (2001). The effects of type I error rate and power of the ANCOVA F test and selected alternatives under nonnormality and variance heterogeneity. J Exp Educ.

[CR61] Schneider IE (2000). Responses to conflict in urban-proximate areas. J Park Recreat Admi.

[CR62] Schneider IE, Arnberger A, Cotrell S, von Ruschkowski E (2019). Modelling impacts of bark beetle infestations on forest visitor experiences and intended displacement. Sci.

[CR63] Schlueter A, Schneider IE (2016). Emerald ash borer management: visitor acceptance and confidence. Sci.

[CR64] Soper DS (2020) A-priori Sample Size Calculator for Structural Equation Models [Software]. http://www.danielsoper.com/statcalc

[CR65] Stedman RC (2003). Is it really just a social construction? The contribution of the physical environment to sense of place. Soc Nat Resour.

[CR66] Stedman RC, Beckley T, Wallace S, Ambard M (2004). A picture and 1000 words: using resident-employed photography to understand attachment to high amenity places. J Leis Res.

[CR67] Stewart WP, Williams DR, Kruger LE (2013). Place-based conservation: perspectives from the social sciences.

[CR68] Steyn P (2013) Data Assumption: homogeneity of variance. https://www.introspective-mode.org/assumption-homogeneity-variance-univariate/

[CR69] Steiger JH, Lind J (1980) Statistically-based tests for the number of common factors. Paper presented at the Annual Spring Meeting of the Psychometric Society. University of British Columbia, Iowa City, IA

[CR70] Stokols D, Shumaker S, Harvey J (1981). People and places: A transactional view of settings. Cognition, social behavior and the environment.

[CR71] Tuan Y (1977). Space and place: The perspective of experience.

[CR72] Twigger-Ross CL, Uzzell DL (1996). Place and identity processes. J Environ Psychol.

[CR73] USDA Forest Service (1985). ROS users guide: Eastern region supplement.

[CR74] Verdin GP, Lee ME, Chavez DJ (2008). Planning forest recreation in natural protected areas of southern Durango, Mexico. Madera y Bosques.

[CR75] Warzecha CA, Lime DW (2001). Place Attachment in Canyonlands National Park: visitors’ assessment of setting attributes on the Colorado and Green Rivers. J Park Recreat Admi.

[CR76] Wilhelm Stanis SA, Schneider IE, Shinew KJ, Chavez DJ, Vogel M (2009). Physical activity and the recreation opportunity spectrum: Differences in important site attributes and perceived constraints. J Park Admi.

[CR77] Williams DR (2007) Recreation settings, scenery, and visitor experiences: a research assessment. In: Kruger LE, Mazza R, Lawrence K (eds) Proceedings: National workshop on recreation research and management. Gen Tech Rep PNW -698. USDA Forest Service, Pacific Northwest Research Station, Portland, OR, pp 29–41

[CR78] Williams DR, Roggenbuck JW (1989) Measuring place attachment: Some preliminary results. Paper presented at the Session on Outdoor Planning and Management, NRPA Symposium on Leis Research. USDA Forest Service, San Antonio, TX, 20–22

[CR79] Williams DR, Patterson ME, Roggenbuck JW, Watson AE (1992). Beyond the commodity metaphor: examining emotional and symbolic attachment to place. Leis Sci.

[CR80] Williams DR, Patterson ME, Roggenbuck JW, Watson AE (1992). The variability of user-based social impact standards for wilderness management. Sci.

[CR81] Williams DR, Stewart SI (1998). Sense of place: an elusive concept that is finding a home in ecosystem management. J.

[CR82] Williams DR, Vaske JJ (2003). The measurement of place attachment: Validity and generalizability of a psychometric approach. Sci.

[CR83] Wollmuth DC, Schomaker JH, Merriam LC (1985). River recreation experience opportunities in two recreation opportunity spectrum (ROS) classes. J Am Water Resour Assoc.

[CR84] Wynveen CJ, Connally WD, Kyle GT (2013). Pro-environmental behavior in marine protected areas: the cases of the Great Barrier Reef Marine Park and the Florida Keys National Marine Sanctuary. J Park Recreat Admi.

[CR85] Wynveen CJ, Kyle GT, Hammitt WE, Absher JD (2008) Exploring the effect of experience use history and place bonding on resource substitution. In: LeBlanc C, Vogt C (eds) Proceedings of the 2007 northeastern recreation research symposium; 2007 April 15-17; Bolton Landing, NY. Gen Tech Rep NRS-P-23. USDA Forest Service, Northern Research Station, Newtown Square, PA, pp 114–122

[CR86] Wynveen CJ, Kyle GT, Sutton SG (2010). Place meanings ascribed to marine settings: the case of the Great Barrier Reef Marine Park. Leis Sci.

[CR87] Wynveen CJ, Kyle GT, Sutton SG (2012). Natural area visitors’ place meaning and place attachment ascribed to a marine setting. J Environ Psychol.

[CR88] Wynveen CJ, Kyle GT, Theodori GL (2010). Place bonding and trust: the case of feral hog management surrounding Big Thicket National Preserve. J Rural. Soc Sci.

[CR89] Wynveen CJ, Schneider IE, Arnberger A (2018). The context of place: issues measuring place attachment across urban forest contexts. J.

[CR90] Wynveen CJ, Schneider IE, Cottrell S, Arnberger A, Schlueter AC, Von Ruschkowski E (2017). Comparing the validity and reliability of place attachment across cultures. Soc Nat Resour.

[CR91] Yamaki K, Shoji Y (2004) Classification of trail settings in an alpine national park using the Recreation Opportunity Spectrum approach. In: Sievänen T et al (eds) Policies, methods and tools for visitor management: Proceedings of the Second International Conference on Monitoring and Management of Visitor Flows in Recreational and Protected Areas, Rovaniemi. Finnish Forest Research Institute, Finland

